# Extensions of *ℓ*_1_ regularization increase detection specificity for cell-type specific parameters in dynamic models

**DOI:** 10.1186/s12859-019-2976-1

**Published:** 2019-07-16

**Authors:** Pascal Dolejsch, Helge Hass, Jens Timmer

**Affiliations:** 1 Institute of Physics, University of Freiburg, Hermann-Herder-Str. 3, Freiburg, 79104 Germany; 2 Signalling Research Centres BIOSS and CIBSS, Schänzlestr. 18, Freiburg, 79104 Germany; 3 Centre for Systems Biology (ZBSA), Habsburgerstr. 49, Freiburg, 79104 Germany

**Keywords:** Systems biology, Dynamic models, Regularization, Feature selection, Accuracy, Sensitivity

## Abstract

**Background:**

Ordinary differential equation systems are frequently utilized to model biological systems and to infer knowledge about underlying properties. For instance, the development of drugs requires the knowledge to which extent malign cells differ from healthy ones to provide a specific treatment with least side effects. As these cell-type specific properties may stem from any part of biochemical cell processes, systematic quantitative approaches are necessary to identify the relevant potential drug targets. An *ℓ*_1_ regularization for the maximum likelihood parameter estimation proved to be successful, but falsely predicted cell-type dependent behaviour had to be corrected manually by using a Profile Likelihood approach.

**Results:**

The choice of extended *ℓ*_1_ penalty functions significantly decreased the number of falsely detected cell-type specific parameters. Thus, the total accuracy of the prediction could be increased. This was tested on a realistic dynamical benchmark model used for the *DREAM6* challenge. Among Elastic Net, Adaptive Lasso and a non-convex *ℓ*_*q*_ penalty, the latter one showed the best predictions whilst also requiring least computation time. All extended methods include a hyper-parameter in the regularization function. For an Erythropoietin (EPO) induced signalling pathway, the extended methods *ℓ*_*q*_ and Adaptive Lasso revealed an unpublished alternative parsimonious model when varying the respective hyper-parameters.

**Conclusions:**

Using *ℓ*_*q*_ or Adaptive Lasso with an a-priori choice for the hyper-parameter can lead to a more specific and accurate result than *ℓ*_1_. Scanning different hyper-parameters can yield additional pieces of information about the system.

## Background

Describing processes in biological systems by mathematical models is a key feature to understand how living organisms work [1]. This task is frequently approached by mechanistic modelling via ordinary differential equations (ODEs). Yet, a severe obstacle to make predictions based on the models consists in the high-dimensional parameter spaces that quickly arise in realistic systems. The steadily growing availability of data and the development of experimental techniques need to be accompanied by statistical methods that can efficiently incorporate them into models even for hundreds of parameters to estimate.

If, for example, two cell types are examined with respect to the same process, additional parameters must be incorporated to describe the second cell type. One may however assume that the cell types of interest differ only in *some* aspects. This assumption would allow to assign some parameters to both cell-types whilst pinpointing the biological differences between the cell-types. In addition, reducing the dimension of the parameter space eases calculations. This idea of selecting only those features that relevantly contribute to the observations can be accomplished by various approaches. The most intuitive way might be to test all possible model configurations iteratively [2, 3]. As for *n* parameters the number of models to test is given by 2^*n*^, this becomes infeasible even for small models.

Whenever the general estimation procedure consists of minimizing an objective function such as the negative log-likelihood, which is equivalent to maximizing the likelihood, *regularizing* the objective function can be regarded as the consequent extension to incorporate equalities among different cell types. Regularization generally refers to including additional information, which here means to amend the objective function by a term which is larger than zero whenever parameters differ between the two cell types. Thus the optimization tends to shrink the model by preferring parameters that are equal among the two cell types. In the context of minimizing a sum of squares, it is intuitive to add a squared function to the objective function that is minimal if the two cell-types behave equally. This can be scaled by a factor *λ*. Then, both functions are minimized simultaneously. This idea has been known as *Ridge Regression* or *Tikhonov Regularization* for many decades now [4–7]. By this method, the minimum of the regularized objective function however only converges asymptotically to a point where some parameters are cell-type independent with increasing penalty strength. Hence, this method does not provide an effective model shrinkage.

With Tibshirani’s introduction of the *Lasso*, i.e. least absolute shrinkage and selection operator, [8], selecting features and estimating optimal parameters was established for linear regressions. The method relies on the *ℓ*_1_ norm of the parameter vector. As this quantity is continuous, it eases numerical calculations. It is however not differentiable if one parameter is zero. If the tuning parameter *λ* is sufficiently large, it enforces a sparse solution [9]. The original Lasso procedure has been generalized: Among others, Adaptive Lasso [10] and Elastic Net [11] have been proposed as they provide better convergence properties or an algorithmic simplification when compared to the original Lasso. In the framework of linear regression, also non-convex penalty functions have been proposed, such as the *ℓ*_*q*_ pseudo-norm of the parameter vector [12].

An adoption, the so-called *ℓ*_1_ regularization, has been used in Systems Biology for non-linear parameter estimation in cellular signalling models [13, 14]. Therefor, the parameters $p_{i}^{[0]}$ of one cell-type are chosen as reference. The parameters of the second cell-type $p_{i}^{[1]}$ are then expressed as the product of fold-changes $\varrho _{i}^{[1]}$ and the reference value $p_{i}^{[0]}$. The penalty term only acts on the logarithmic fold-changes. This method finds relevant differences between two cell types. These particularities of one cell-type could serve as targets for drugs that shall only affect malign cells [13]. However, Steiert et al. [14] point out that the amount of properties which are falsely detected as cell-type specific can be decreased by manual supervision of the regularization outcome. Hence, it is desirable to find a penalization that requires no manual checking to find potential drug targets in a more robust way.

One major limitation to *ℓ*_1_ regularization is the presence of linearly correlated log-transformed parameters. Consider a reaction that involves the product of two kinetic rates *p*_1_×*p*_2_ or their quotient. A log-transformation can then lead to a linear functional relation between the estimated values that minimize the objective function: $\log \hat p_{1}=\mathrm {const.}\pm \log \hat p_{2}$. Both parameters may still be identifiable if they appear independently in other reactions, too. Thus, the linear correlation does not hamper the optimization process if only one cell-type is modelled. When including a second cell-type, the linear relation however translates to a linear correlation between the log-fold-change estimates:

1 $$ \log\hat\varrho_{1}=\mathrm{const.}\pm \log\hat\varrho_{2}.   $$

**Fig. 1:**
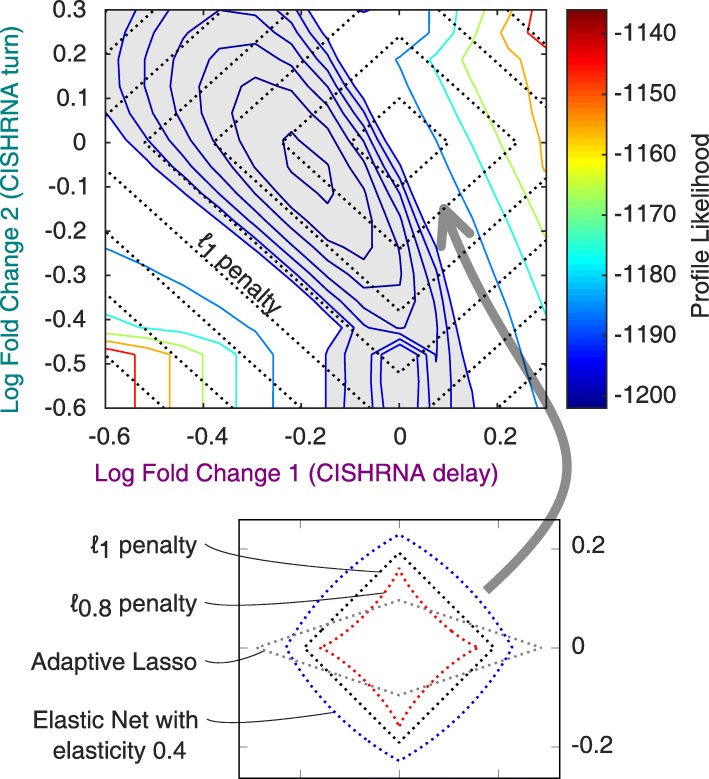
Level lines of objective function and penalty terms. Upper panel: Two-dimensional Profile Likelihood for two linearly correlated log-fold-changes, taken from an EPO-induced JAK2/STAT5 signalling pathway [13, 15]. The dashed lines indicate the level lines of an *ℓ*_1_ penalty. The gray area marks the 95*%* confidence region. Lower level: Two-dimensional level lines of constant penalty for the *ℓ*_1_/Lasso penalty function and the three extended methods presented in this manuscript: Non-convex *ℓ*_*q*_, Adaptive Lasso and Elastic Net penalty. The extended methods can replace the *ℓ*_1_ penalty in the upper panel, which is analyzed in the “Results” section. The level lines of *ℓ*_1_ are aligned to the gray confidence region, where the extended methods produce a deformation in the level lines that can prevent an alignment

Figure 1 shows an example of linearly correlated log-fold-changes taken from an EPO induced JAK2/STAT5 signalling pathway [13, 15]. The model will be discussed in detail below. The objective function landscape reveals a minimum. However, the surrounding confidence region is aligned to the diagonal subspace $\log \hat \varrho _{1}=\mathrm {const.}-\log \hat \varrho _{2}$ for the two fold-change parameters belonging to the CISHRNA *turn* and *delay* rates, respectively. If *ℓ*_1_ penalization is applied to such parameters, the space of constant penalty also partially coincides with the diagonal subspace. The *ℓ*_1_ penalty does not provide additional degrees of freedom apart from the linear scaling factor *λ*. Hence, the co-alignment of penalty and confidence region cannot be prevented, so *ℓ*_1_ can be considered as too *rigid* in this case. The linear relation has to be distinguished from a structural non-identifiability of the model itself (cf. [16]). Eq. 1 appears within the introduced penalty term while the model parameters themselves remain identifiable.

Here, we propose the use of extended *ℓ*_1_ methods—Adaptive Lasso, Elastic Net and the non-convex *ℓ*_*q*_ penalty—to achieve a more flexible regularization technique. Each of these methods introduces an additional degree of freedom to make the set of available penalty functions less rigid than only *ℓ*_1_. This *deformation* degree of freedom determines, how much the level lines differ from those of *ℓ*_1_. The shapes of the level lines corresponding to these extended penalty functions for exemplary deformation parameters are depicted in Fig. 1. If the extended penalty terms are added to the objective function, the deformation parameter can be tuned by varying the additional degree of freedom, so the level lines of the penalty do not coincide any more with the alignment of the objective function. Hence, a shrinkage can be re-established. All methods presented in this manuscript are available within the MATLAB modelling framework *Data2Dynamics* [17, 18]. This paper aims to extend the possibilities described by Steiert et al. [14] and Merkle et al. [13]. Thus, an introduction to the already published idea of *ℓ*_1_ regularization and of the optimization routine are given first. In a second step, the extended methods, which have not been used in a non-linear dynamic modelling setting to our knowledge, are introduced and discussed. The accuracy of the prediction of cell-type dependent parameters could be significantly increased when using Adaptive Lasso or *ℓ*_*q*_ penalties because these methods could reduce the number of falsely detected cell-type specific parameters.

## Methods

### Parameter estimation

Biological processes in cells can be translated into a system of coupled ODEs with the concentrations being time-dependent functions:

2 $$ \dot{{x}}(t) = f\left({x}(t),u(t,p_{u}),p_{x}\right), x(0) = p_{0},   $$

where *x* denotes the system’s intrinsic state, *u* a possibly existing external input, *p*_*u*_,*p*_*x*_ a set of parameters, *p*_0_ the initial conditions and *f* a continuous function that is determined by the biological properties of the system. All quantities are considered as vector valued.

The internal states *x* that solve Eq. 2 are usually not accessible to an experimental observer, so all measurable quantities *y* are mappings from the space of internal states onto the observer space

3 $$ y(t) = g\left(x(t),p_{y}\right) + \epsilon(t)   $$

with some observation parameters *p*_*y*_ and a measurement error $\epsilon (t)\sim \mathcal {N}\left (0,\sigma ^{2}(p_{\sigma })\right)$. The latter will be assumed as normally distributed although the approach is not limited to this case. The observation function *g* depends on the observational set-up. The states *x*(*t*) and *y*(*t*) will be considered as vector-valued. The functions *f* and *g* are known except for the parameter values, so a set of parameters

4 $$ p = (p_{0},p_{x},p_{y},p_{u},p_{\sigma}),   $$

which is assumed as constant in time, is necessary to completely characterize a system as described in Eqs. (2) and (3).

All equations are assumed to have only positive parameters. The parameters will be estimated on a logarithmic scale, also in order to avoid numerical instabilities among different orders of magnitude of the parameters. It can be shown that observables in biological processes are usually log-normally distributed [19], so the uncertainties of their log-transform follow a Gaussian distribution.

**Maximum likelihood approach** Given data points *y*_*ij*_=*y*_*i*_(*t*_*j*_) for *M* states *y*_*i*_ and *N* time points *t*_*j*_ as well as the corresponding observation function values *g*(*x*_*i*_(*t*_*j*_)) resulting from the ODE system (Eq. 2) and standard deviations *σ*_*ij*_, the negative 2-fold log-likelihood

5 $$ \sum\limits_{i,j=1}^{M,N}\left(\!\frac{y_{{ij}}-g(x_{i}(t_{j}))}{\sigma_{{ij}}}\!\right)^{2}\,=\,\mathrm{const.}-2\log\mathcal{L}(p)=:\chi^{2}_{\text{ML}}(p)   $$

is minimized. This yields the maximum likelihood parameter estimate $\hat p^{\text {ML}}=\arg \min _{p}\chi _{\text {ML}}^{2}(p)$. In cases of unknown *σ*_*ij*_, additional terms must be taken into account [20].

To optimize the likelihood $\chi ^{2}_{\text {ML}}$ (Eq. 5), it is generally necessary to apply numerical methods because no analytic solutions are available.

**Profile likelihood** The profile likelihood $\chi ^{2}_{\text {PL}}(p_{i})$ is obtained by re-optimizing the objective function for each value of *p*_*i*_ with respect to all remaining parameters *p*_*i*≠*j*_ [16, 21, 22]:

6 $$ \chi^{2}_{\text{PL}}(p_{i}) = \min_{p_{j\neq i}}\chi_{\text{ML}}^{2}(p_{j}).  $$

By calculating the profile likelihood for each parameter, the confidence interval $\text {CI}(\hat p)$ around an optimum $\hat p$ can be determined:

7 $$ \text{CI}(\hat p) = \left\{p\mid\chi_{\text{ML}}^{2}(p)-\chi_{\text{ML}}^{2}\left(\hat p\right)< q_{\alpha}^{(m)}\right\},  $$

where $q_{\alpha }^{(m)}$ denotes the *α*-quantile of the *χ*^2^ distribution with *m* degrees of freedom. For *α*=0.95, the threshold is $q_{0.95}^{(1)}=3.84$ for determining confidence intervals for one parameter.

### Regularization for two cell-types

Consider two cell-types [0] and [1]. If the model describes a biological process that both of them may undergo, the ODE system (Eq. 2) does not require changes, whereas the parameter values of *p* can depend on the cell-type. This section recapitulates the basic denotions as described by Steiert et al. [14].

**Log-fold-changes** Subsequently, one cell-type will be chosen as reference, corresponding to a parameter set *p*^[0]^. For the other cell-type, only the fold-changes *ϱ*^[1]^ with respect to the type of reference will be considered, which are defined as

8 $$\begin{array}{*{20}l} \varrho_{i}^{[1]} = \frac{p_{i}^{[1]}}{p_{i}^{[0]}} {}\Leftrightarrow{} r_{i}^{[1]}:=\log\varrho_{i}^{[1]}=\log p_{i}^{[1]} - \log p_{i}^{[0]}.  \end{array} $$

with the log-fold-change vector *r*:=*r*^[1]^, which will be used as only the log transformations of parameters *p* are considered. Thus, $r_{i}^{[1]}$ is zero if and only if the value of parameter $p_{i}^{[1]}$ of cell-type [1] is compatible with $p_{i}^{[0]}$, associated with the type of reference [0]. This parameter may then be called *cell-type independent*, while the term *cell-type specific* refers to the opposite case, $r_{i}^{[1]}\neq 0$.

**Penalization** If both cell types are likely to share certain properties, it can be assumed that some fold-change parameters *r*_*i*_ vanish. Hence, the model is supposed to be *sparse* with respect to *r*. To incorporate this prior knowledge, the original objective function $\chi ^{2}_{\text {ML}}$ is amended by a penalty term *ν*(*r,r*^∗^):

9 $$ \chi^{2}(p,r,r^{*},\lambda) = \chi^{2}_{\text{ML}}(p,r) + \lambda\,\nu(r,r^{*}),\quad\lambda\geq0,   $$

where the function *ν* only depends on fold-change parameters and has its global minimum at a target value *r*^∗^. For a sparse two-cell-type model with logarithmic parameters, *r*^∗^=0 is chosen. Other values of *r*^∗^ might be useful in cases where assumptions other than model sparsity motivate the usage of regularization. The dimensionless tuning parameter *λ* determines the penalization strength. Hence if *λ* is chosen large enough, the penalty should enforce that *r*=0 be a solution to the optimization problem. This approach differs from the original *Lasso* regularization as it only penalizes the fold-changes, i.e. the subset of all parameters that links cell-type [1] to the cell of reference [0].

**Two-step regularization routine** All parameter estimates resulting from the optimization of a penalized objective function (Eq. 9, *λ*>0) must be considered as possibly biased by the penalty term. To circumvent this problem, a two-step estimation routine is implemented in Data2Dynamics and was used throughout all presented calculations.

1. Optimize the penalized objective function (Eq. 9) to obtain the subset of zero log-fold-changes *Z*(*λ*) for a given penalty strength *λ*>0.
2. Remove the regularization (*λ*=0) and set those parameters belonging to *Z*(*λ*) as fixed to zero. Then re-optimize the unbiased objective function.

The penalty strength *λ* essentially determines how many parameters are set to zero. Therefore, several orders of magnitude are scanned for *λ*, and for each of them the aforementioned two-step estimation is executed. To determine the sparsest model that can still be considered as consistent with the data, which will be referred to as *parsimonious model*, information theory based criteria have been developed. The likelihood ratio test (LRT, [23]), the Akaike information criterion (AIC, [24]) and the Bayesian information criterion (BIC, [25]) are the most prominent ones. As AIC generally selects too large models, it is not a consistent selection criterion. Since BIC is equivalent to LRT for an adjusted threshold *α*, only LRT will be considered here to find the parsimonious model in order to allow for a comparison to the *ℓ*_1_ results found by [14] when using LRT.

After performing the two-step estimation, the final objective function value depending on *λ* is given by

10 $$ \chi^{2}(\lambda)=\chi^{2}_{\text{ML}}(\hat p^{\text{ML}},\hat r^{\text{ML}}(Z(\lambda))).  $$

From this, the LRT statistic *D*(*λ*) is defined as

11 $$ D(\lambda)=\chi^{2}(\lambda)-\chi^{2}(0),  $$

quantifying the objective function difference to a not-regularized model (*λ*=0). Given a statistical significance level *α*, the model shrinkage induced by a penalty with strength *λ* is considered compatible with the data if *D*(*λ*) does not exceed $q_{\alpha }^{\#Z(\lambda)}$. This is the *α* quantile of a *χ*^2^ distribution with *#Z*(*λ*) degrees of freedom. The *parsimonious model*, which has least cell-type specific features among all models that agree with data up to the *α* level, is found at the optimal penalty strength

12 $$ \lambda^{*}=\max\left\{ \lambda>0\mid Z(\lambda)=\emptyset\text{ or}\ D(\lambda)< q_{\alpha}^{\#Z(\lambda)}\right\}.   $$

The first condition in Eq. (12) is necessary to include also those penalty strengths which do not lead to shrinkage.

### Extended penalty functions for regularization

**Fig. 2:**
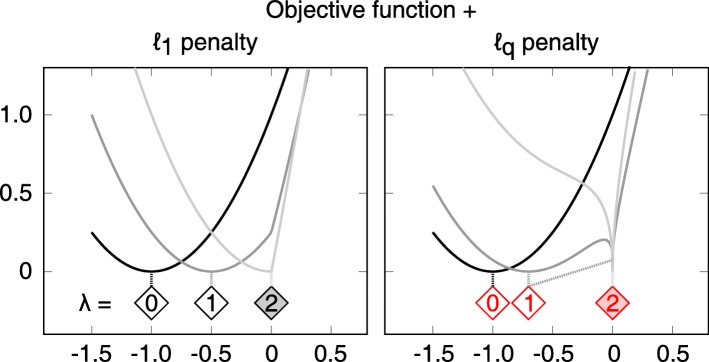
Non-smooth penalties shift the objective function minimum to zero. Shift of the objective function minimum towards zero for increasing penalty strength. The horizontal positions of the diamond tips mark the global minimum of the regularized objective function with the penalty strength denoted inside. The black curves represent an unpenalized objective function $\chi ^{2}_{\text {ML}}$. Dark-gray curves depict the sum of $\chi ^{2}_{\text {ML}}$ and a penalty with strength *λ*=1. The filled diamonds in zero represent penalty strengths *λ*_*Z*_ which cause the minimum to be exactly in zero. The objective function penalized with *λ*_*Z*_ is drawn in light grey. Finite *λ*_*Z*_=2 is sufficient to shift the minimum to zero. While the convex absolute-value penalty only admits one minimum, the non-convex *ℓ*_*q*_ penalty can lead to multiple local minima as depicted for *λ*=1 on the right hand panel. For most optimizations described in this manuscript, an *ℓ*_0.8_ penalty was used. In this figure, the case of *q*=0.5 is depicted to show the multiple minima more clearly. They arise, however, for any 0<*q*<1

The original log-likelihood is a sum over squares, so defining $\nu (r,r^{*}=0)=\sum \nolimits _{i}r_{i}^{2}$ would be a consequent approach. The smoothness of *ν* in *r*^∗^=0 however leads to only asymptotic optimality of the sum of objective function and penalty term in *r*=0 for *λ*→*∞* [9]. If the sum over absolute values is chosen, $\nu (r)=\sum \nolimits _{i}|r_{i}|$, the model can be shrunk even for finite *λ*, i.e. *r*_*i*_=0 for some *i* in the optimum of *χ*^2^, see Fig. 2. This is due to the non-differentiable point of *ν* whenever *r*_*i*_=0 for some *i*. The effect of this so-called *ℓ*_1_ penalty was studied both, for simulated [14] and experimental data [13].

Choosing a penalty

13 $$ \nu(r)=\sum\limits_{i} |r_{i}|^{q},   $$

with 0<*q*<1 still implies a non-differentiability of *ν* if *r*_*i*_=0 for any *i*. Moreover, this function is not convex in the case *q*<1. Although [9] postulated that this would cause severe problems for the optimization routine, [12] showed that non-convex penalty functions can lead to better results compared to *ℓ*_2_ or *ℓ*_1_ in linear cases.

The gradient of the *ℓ*_*q*_ penalty function (Eq. 13) reads

14 $$ \nabla\nu(r)_{i} = q\,|r_{i}|^{q-1}\,\text{sign}(r_{i})  $$

if *r*_*i*_≠0. This term diverges for *r*_*i*_→0, with a right-side limit of +*∞* and a left-side limit of −*∞*. Hence there is always an (at least local) optimum of the total objective function in *r*_*i*_=0, see Fig. 2, right panel. This is independent of the underlying objective function and can be considered as an artefact. To avoid that the optimizer is constrained to this point even if it is not the global optimum, a small *ε*≈10^−10^ is chosen. The optimizer then considers all *r*_*i*_ with |*r*_*i*_|≤*ε* as effectively zero, while the gradient is fixed to

15 $$ \nabla\nu(r)_{i}= q\,\varepsilon^{q-1}\,\text{sign}(r_{i})\text{ for all }i,  $$

representing the gradient value of the limiting case |*r*_*i*_|=*ε* and hence remaining finite.

Two other penalty functions, which had been developed for linear problems, were tested as well. The *Elastic Net*

16 $$\begin{array}{*{20}l} \nu(r)&=(1-\alpha)\sum\limits_{i}|r_{i}|+\alpha\sum\limits_{i}r_{i}^{2}, \end{array} $$

17 $$\begin{array}{*{20}l} \nabla\nu(r)_{i}&=(1-\alpha)\,\text{sign}(r_{i})+2\alpha r_{i}, \end{array} $$

was introduced by [11] to reduce the bias of predictions for elasticities 0≤*α*≤1. This penalty function is strictly convex for *α*>0, but singular for *r*_*i*_=0 for *α*<1. It includes the special cases of *ℓ*_1_ and *ℓ*_2_ penalties for *α*=0 or *α*=1, respectively.

The *Adaptive Lasso*:

18 $$\begin{array}{*{20}l} \nu(r)&=\sum\limits_{i} \lvert r_{i}\rvert\times\left\lvert \hat r_{i}^{\text{ML}}\right\rvert^{-\gamma}, \end{array} $$

19 $$\begin{array}{*{20}l} \nabla\nu(r)_{i}&=\text{sign}(r_{i})\left\lvert \hat r_{i}^{\text{ML}}\right\rvert^{-\gamma}, \end{array} $$

was introduced by [10] given maximum likelihood estimates $\hat r_{i}^{\text {ML}}$ and adaptivities *γ*>0. This approach was proved to asymptotically provide unbiased estimates in the linear setting, keeping a convex penalty function.

The three methods defined above all contain one additional *deformation* parameter *d*∈{1−*q*,*γ*,*α*}. This terminology refers to the effect that they determine how much the level lines of the penalty functions are deformed with respect to the rigid diamond of *ℓ*_1_, see Fig. 1. The limit *d*→0 always yields the original *ℓ*_1_.

All gradients are not defined if *r*_*i*_=0, but sub-differentials can be obtained by defining sign(0)=[−1,1] [26]. This leads to set-valued gradient components in singular points. The method is applicable only to convex functions, so for the *ℓ*_*q*_ penalty, this approach holds only within the small *ε* neighbourhood around zero. There, the gradient modulus is constant, making the penalty effectively behave like the modulus function, hence it becomes convex.

To determine the optimal estimate $(\hat p,\hat r)$ for the objective function *χ*^2^(*p*,*r,r*^∗^=0,*λ*) (Eq. 9) for given *λ*, the following criteria must be fulfilled [14]:

20 $$\begin{array}{*{20}l} \nabla_{p}\chi^{2}(\hat p,\hat r)&{}=0,&&\text{ and for each } {i} \text{ either} \end{array} $$

21 $$\begin{array}{*{20}l} \nabla_{r}\chi^{2}(\hat p,\hat r)_{i}&{}=0,&&\text{ for }|r_{i}|>0\text{, or} \end{array} $$

22 $$\begin{array}{*{20}l} \nabla_{r}\chi^{2}_{\text{ML}}(\hat p,\hat r)_{i}&{}\in\lambda\nabla\nu(\hat r)_{i},&&\text{ for }r_{i}=0. \end{array} $$

The first and second criterion (Eqs. 20 and 21) represent the requirement of vanishing gradients in all non-singular cases. The first and third criterion (Eqs. 20 and 22) are fulfilled if the parameters $\hat p$ are optimal and the penalty term dominates the maximum likelihood contributions. According to subdifferential calculus, this is sufficient to obtain an optimal point [26].

### Implementation

All calculations to optimize the objective functions (Eqs. 5 and 9) are performed within the MATLAB framework Data2Dynamics [17, 18]. This is a freely available, state-of-the-art software package that has been used for various system biology applications [13, 27, 28], performing parameter estimation, uncertainty analysis and prediction calculation. It also contains a toolbox for regularizing models by *ℓ*_1_ or *ℓ*_2_ penalties. Elastic Net, Adaptive Lasso and *ℓ*_*q*_ penalties for arbitrary *q*>0 have been integrated, keeping the already existing structure as described by [14]. The regularization routines can be applied to all models and data types that Data2Dynamics supports. They do not pose any restrictions such as normalization or an absolute scale on the experimental data that is used for modelling. Yet, the complexity of the underlying mathematical model should be tailored to the information available in the data, c.f. [29]. Further details on the usage of the new methods are given in the main regularization routine arRegularize, which can be found at the directory arFramework3/L1/arRegularize.m within Data2Dynamics.

## Results

### Application on simulated data

In a first step, a simulation study is employed to investigate in how far Elastic Net, Adaptive Lasso or non-convex *ℓ*_*q*_ penalties can lead to an improved estimation compared to the already established *ℓ*_1_ approach. The *M1* model from the sixth Dialogue for Reverse Engineering Assessment and Methods challenge (DREAM6, [30]) was used because it provides comparability with the results of pure *ℓ*_1_ regularization obtained by [14].

**Model description** The model system is composed of six genes, for which the concentrations of protein and mRNA were incorporated. The translation rate for the synthesis of proteins was considered as proportional to the concentration of mRNA and the strengths of the ribosomal binding sites. The transcription rates were assumed to follow Hill kinetics. Since simulated data was used for this model, no units for time and concentrations were specified. For the challenge, all mRNA degradation rates were assumed to be equal to 1 inverse time unit. This results in a total number of 29 kinetic parameters with six ribosomal and six protein synthesis strengths, one degradation parameter, eight *K*_*m*_ values and eight Hill coefficients [14, 30]. The full description of the model and the parameter values is available from the DREAM6 organizers under www.synapse.org/#!Synapse:syn2841366/wiki/71372. It is also included as an example in the Data2Dynamics framework.

The model was simulated 150 times for two cell-types. The first cell-type, which was used as reference for the fold-changes of the second, corresponded to the gold standard parameter set of the DREAM6 challenge in the first place. The Hill coefficients range from one to four which represents the number of ribosomal binding sites. For the second cell-type, fold-change parameters were introduced to relate the parameter values of both cell-types. For each simulation run independently, approximately one third of the 29 dynamic parameters were chosen as cell-type specific. The respective fold-changes were then drawn randomly from the set *ϱ*∈{1/10,1/5,1/2,2,5,10} for the non-Hill parameters. Fold-changes for Hill parameters were randomly selected as *ϱ*∈{1/4,1/2,2,4} such that the parameter values of both cell-types are within the interval [1,4].

By additionally introducing perturbations to the kinetic rates of one gene, in total experimental 18 set-ups were available: First, each gene could be knocked out, so it would not be produced at all. Second, a mRNA degradation rate could be increased by a factor of five for one gene. Third, the mRNA synthesis rate of one gene could be doubled. For each run, it was randomly selected whether each of these additional set-ups was observed.

It was assumed that either mRNA or proteins were observed to account for only partial observability in real-world models. The mRNA observation had a probability to be selected of one third. In this case, 21 data points of each mRNA would be observed. In the remaining two thirds of the cases, two selected proteins were measured with 41 data points each. Thus it was random whether the perturbation occurred in the variables that were observed or not.

In total, the resulting 150 simulation runs differed randomly with respect to the cell-type specific parameters, the magnitude of the corresponding fold-change, the available experimental set-ups and the observable quantities. For a more detailed set-up description, the reader is referred to [14]. When applying the regularization techniques as described above, the goal was to test in how far the algorithm would be able to find the cell-type specific features of each of the 150 models that were used as input.

**Fig. 3:**
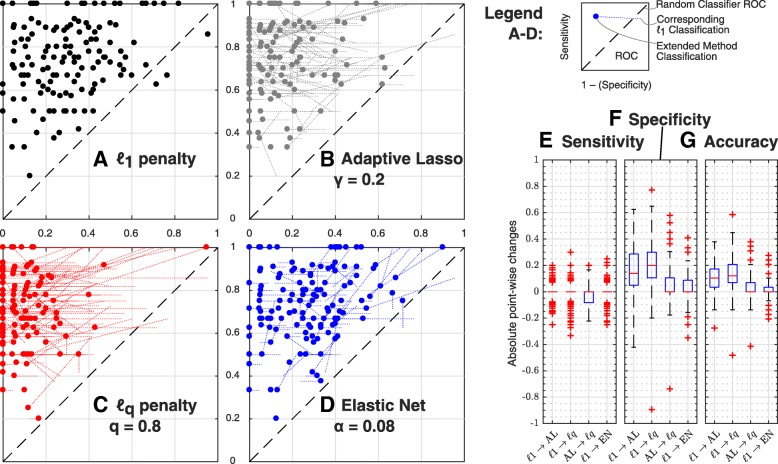
ROC data and accuracies for DREAM6, Model 1. **a–d**: ROC data for the four penalty functions applied: *ℓ*_1_, Adaptive Lasso, *ℓ*_0.8_ and Elastic net. The diagonal, black dashed line represents the characteristic that can be expected from a random classifier. The upper left corner of each ROC curve represents the optimal classification. The thin dotted lines show the changes with respect to the same run penalized by *ℓ*_1_. Mean curves were omitted to focus on the differences between *ℓ*_1_ and the extended methods. Both, Adaptive Lasso and *ℓ*_0.8_ lead to mostly horizontal changes compared to *ℓ*_1_, which corresponds to an increased specificity and a constant sensitivity. **e–g**: Absolute run-wise changes for extended methods vs. *ℓ*_1_ and Adaptive Lasso against *ℓ*_*q*_. Boxplot whiskers extend to twice the interquartile range at most. The boxplots emphasize the gain in specificity and consequently in accuracy

**Results of*ℓ***_**1**_** regularization** For 150 repetitions of the above mentioned random model and observation calculations, the parsimonious model was calculated by applying *ℓ*_1_ regularization to all fold-change parameters. Therefore, the penalty strengths were scanned from *λ*=10^−4^ to *λ*=10^6^. The parsimonious model was determined by virtue of Eq. (12). Then, the regularization result whether a fold-change was non-zero (called *positive* prediction) was compared to the true parameter values used to simulate the data. An overall accuracy (correct over total classifications) of (71 ± 13)*%* was obtained, which is in accordance with the overall result of 78*%* and also with the accuracies sorted by parameter type, published by [14]. A receiver operating characteristics (ROC) plot is depicted in Fig. 3a. The average sensitivity (correct over total positive classifications) of (74 ± 17)*%* and the average specificity (correct over total negative classifications) of (70 ± 19)*%* also match with the previously published values of 74*%* and 80*%*, respectively.

**Results of extended penalty functions** Non-convex *ℓ*_0.8_ penalty, Adaptive Lasso (with deformation *d*=0.2) and Elastic Net (*d*=0.08) were applied to the same settings as *ℓ*_1_. The corresponding ROC curves are shown in Fig. 3b–d together with the *ℓ*_1_ result, visualizing that for many runs, the false positive rate was decreased to a large extent (mostly horizontal dotted lines). The point-wise absolute change *Δ* was calculated for sensitivity, specificity and accuracy to compare all extended methods with *ℓ*_1_ and Adaptive Lasso with *ℓ*_*q*_ in addition. Box plot diagrams of *Δ* are depicted in Fig. 3e–g. The mean values $\bar \Delta $ are given in Table 1. A *t*-test has been performed with the null hypothesis of $\bar \Delta =0$. Only changes |*Δ*|>0.1 will be considered as relevant.

**Table 1:** Differences in sensitivity, specificity and accuracy between the presented penalty functions

Methods	$\bar \Delta $ Sensitivity	$\bar \Delta $ Specificity	$\bar \Delta $ Accuracy
*ℓ*_1_→AL	N.S.	+0.16 (***)	+0.11 (***)
*ℓ*_1_→*ℓ*_*q*_	N.S.	+0.21 (***)	+0.13 (***)
AL →*ℓ*_*q*_	−0.025 (**)	+0.052 (***)	+0.026 (*)
*ℓ*_1_→EN	N.S.	+0.032 (**)	+0.020 (**)

Mean absolute point-wise changes $\bar \Delta $ of sensitivity, specificity and accuracy for the presented methods. The total significance level of 5*%* has been corrected by the Bonferroni method to 5*%*/12=0.42*%*

^(*):^ *p*<0.42*%*

^(**):^ *p*<0.042*%*

^(***):^ *p*<0.0042*%*

N.S.: not significantly different from zero

The sensitivity could not be increased significantly by any of the new methods compared to *ℓ*_1_. In turn, both, Adaptive Lasso and *ℓ*_*q*_ were able to significantly increase the specificity. The mean improvements of 0.16 and 0.21, respectively, are also relevant as they reduce the false positive rate from initially 30*%* to only 9*%* and 14*%*, shrinking the number of falsely detected cell-type dependencies to a third or at least one half compared to *ℓ*_1_.

The total accuracy is hence increased significantly by all methods, with Adaptive Lasso and *ℓ*_*q*_ providing the largest improvements. The *ℓ*_*q*_ penalty provides more accurate results than Adaptive Lasso, yet the improvement is considered as minor.

**Fig. 4:**
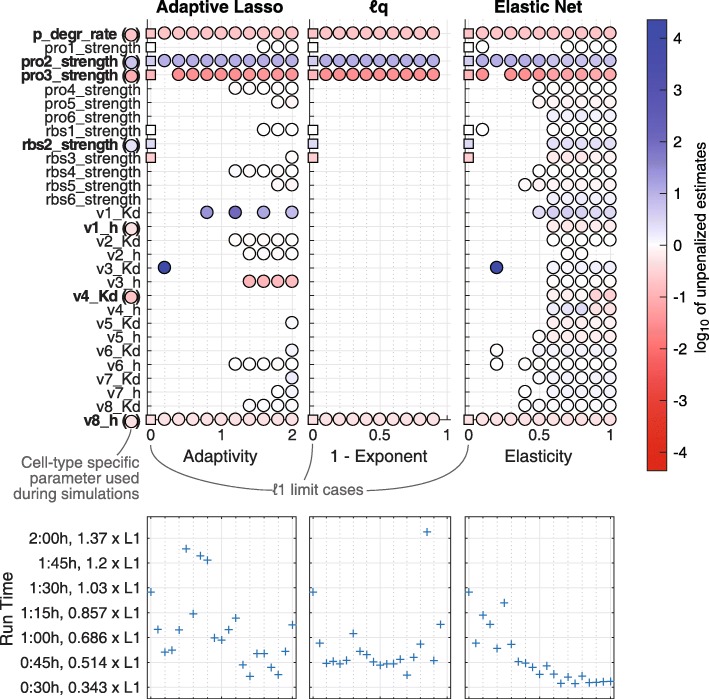
Parsimonious models for some choices of deformation parameters. Upper row: Parsimonious models by scanning over penalty strengths *λ* for a fixed deformation parameter. Only the log-fold-change parameters are shown. Filled circles depict the value of those log-fold-changes that are non-zero after regularization. Squares denote the *ℓ*_1_ penalty at zero deformation, while circles represent the extended functions. Kinetic rates in bold face were chosen as cell-type specific for data simulation. The colours of circles after these kinetic rate names encode the corresponding value. For *ℓ*_*q*_, the parsimonious model was independent of the choice of *q*. Elastic Net did not induce an effective model shrinkage for *α*>0.5. Lower row: The total effective run times of the regularization routines as function of the deformation. These account for the entire two-step regularization routines to find the parsimonious models. All runs were executed on a 20-core server

**The influence of the deformation parameter** The *ℓ*_1_ penalty function has no degree of freedom, so it cannot be adjusted when finding likelihoods aligned along the penalty level lines. To circumvent this, a deformation parameter *d* was used in *ℓ*_*q*_, Adaptive Lasso and Elastic Net penalties. As *d* does not carry a biological meaning, the choice of *d* can be regarded as arbitrary. Therefore, different values were tested for one configuration containing seven cell-type specific features: the protein degradation rate, two protein and one ribosomal synthesis strengths, two Hill exponents and one *K*_*d*_ value. As deformations, adaptivities *γ*∈ [0,2], exponents *q*∈(0,1] and elasticities *α*∈ [0,1] were applied. The results are depicted in Fig. 4. The specificity of the *ℓ*_1_ regularization was 19/22=86.4*%*. Interestingly, *ℓ*_*q*_ penalties yield a unique result independent of *q*<1 with a specificity of 100*%*. Hence, *ℓ*_1_ should not be considered as a strict mathematical limiting case for *q*→1, but rather a different class of penalty function. This might be related to the fact that the penalty term becomes convex for *q*=1. Adaptive Lasso in turn shows varying shrinkage. Especially for adaptivities *γ*>1, the false positive detection increases although the final estimates have small absolute values. For elasticities *α*>0.5, the Elastic Net provides hardly any shrinkage. This demonstrates that the penalty is then closer to Ridge Regression than to Lasso, hence sparsity is only reached asymptotically.

Some parameters were estimated to be cell-type specific for almost all deformations *d* (protein degradation rate, synthesis strengths of protein 2 and 3, and Hill kinetic exponent 8), all of which are true positive classifications. This shows that the flexibility introduced by the deformation *d* encourages an additional scan over a range of possible values to find stronger evidence for true cell-type specific properties. However, it is not necessary to scan over all admissible deformations to achieve model shrinkage. A regularized optimization can be performed with an a priori choice of for example an *ℓ*_*q*_ penalty with *q*=0.8. This avoids possible numerical instabilities for too small *q*, whereas it is sufficiently different from *ℓ*_1_, too, to exhibit the advantageous features of non-convexity.

None of the applied regularization functions was able to detect the cell-type specific behaviour related to Hill coefficient 1 and *K*_*D*_ parameter 4 except for those Elastic Net configurations that could not effectively shrink the model size. This coincides with previous findings that Hill and *K*_*D*_ coefficients are less frequently detected as cell-type specific compared to the remaining parameters. This occurs because the identifiability of those parameters is limited if the corresponding regulator concentration does not lie around *K*_*D*_, and because they are easily concealed by measurement uncertainties [14].

The lower row of Fig. 4 indicates the effective time that was necessary to detect the parsimonious model for a given deformation. It displays that all scans with extended penalty function were faster than the original *ℓ*_1_ except for four deformation values. This holds even for *ℓ*_*q*_, which was considered as hampering the optimizer due to its nonconvexity [9]. A precise assessment of the efficiency is postponed to further research.

**The influence of the*ℓ***_***q***_** cut-off threshold** The *ℓ*_*q*_ regularization requires a threshold parameter *ε* to cut off the unbounded gradients for zero log-fold-changes. Setting *ε*=0 can hamper the simulation if some log-fold-changes are initialized as zero when finding the parsimonious model because these parameters remain zero independent of the scanned penalty strengths. This behaviour that matches the expectations from theory disappeared for all tested positive values of *ε*, even for *ε*∼10^−16^. To avoid numerical problems with rounding, the threshold was fixed to *ε*=10^−10^.

### Application on biological data

**Fig. 5:**
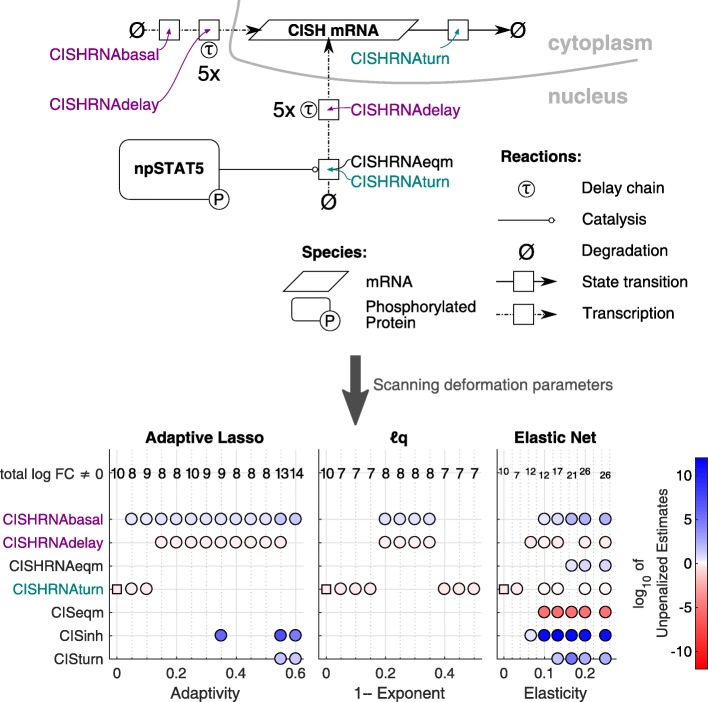
Two parsimonious models for the EPO induced JAK2/STAT5 signalling pathway. Upper panel: Part of the EPO induced JAK2/STAT5 signalling pathway related to the synthesis of CISH mRNA [13]. Dash-dotted arrows denote a transcription, solid arrows a state transition. Boxes represent the kinetic rates associated to a reaction. The delay chain *τ* is modelled with five intermediate steps. The turquoise rate is cell-type specific according to *ℓ*_1_, purple rates are cell-type specific according to some *ℓ*_*q*_ and Adaptive Lasso regularizations. Lower panel: Scan over deformations for the JAK2/STAT5 signalling pathway with CFU-E and H838 cells. Parsimonious Model parameters related to the CISH feedback loop are depicted only. Dot colours represent the final unpenalized estimates. The numbers in the upper row indicate the total number of log-fold-changes not estimated to zero, also including those that are not depicted here. Squares denote the results obtained by the original *ℓ*_1_ penalty. Circles indicate the extended methods. Two possible parsimonious models were found when scanning the deformation strengths: either CISHRNA basal and delay rates were cell-type specific or the corresponding turn rate

The *ℓ*_1_ penalty function has previously been applied to an EPO induced JAK2/STAT5 signalling pathway [13] to find cell-type specific behaviour between healthy CFU-E cells and non-small cell lung cancer cells of type H838. This model contains two feedback loops related to the proteins CISH (see Fig. 5) and SOCS3, both induced by nuclear pSTAT5. 1141 data points are available. For a detailed description, the reader is referred to [13]. By applying an *ℓ*_1_ penalty to the published data, 10 out of 26 kinetic parameters were found to be cell-type specific. Three of these could be removed manually as they were compatible with zero within the confidence interval. The remaining seven cell-type specific properties reproduce the published results.

Scanning over deformations *d* reveals a setting different from the *ℓ*_1_ result for the CISH feedback loop as Fig. 5 reveals. According to the *ℓ*_1_ prediction, only the turnover rate CISHRNAturn is cell-type specific, which is the rate constant for the nuclear pSTAT5 induced synthesis of CISH mRNA. Especially *ℓ*_*q*_ for *q*∈ [0.2,0.35] pinpoints an alternative that considers CISHRNAbasal and CISHRNAdelay as cell-type specific. This corresponds to a basal, not npSTAT5 induced synthesis rate of CISH mRNA and to the delay chain parameter that is applicable to both types of synthesis. The Adaptive Lasso yielded the same classification for some adaptivities. The objective function values of −1197.14 for the turn parameter and −1196.73 for the basal and delay parameters as cell-type specific are almost equal. The profile likelihood of the turn fold-changes, which was presented in the introduction (see Fig. 1), underlines that it would be in accordance with the available data to set this value to zero. Both CISH parameter choices are parsimonious in the sense of Eq. 12. However, the option that considers both, the basal and the delay rate as cell-type specific yields a less sparse result as there remains one parameter more to be estimated. Additional informative data would be required to find better evidence for the cell-type specific properties of the CISH feedback loop. Elastic Net regularization cannot provide informative results that go beyond *ℓ*_1_. Even for low elasticities the shrinking capacity is low.

## Discussion

Systems biology utilizes mathematical models to understand biological processes. Frequently, mechanistic models are built up from ordinary differential equations requiring the estimation of model parameters and a statistical test of the obtained results. A model of biological systems with two cell types can be regularized to force some of the model parameters to be equal amongst the cell types. This induces a sparse system with a reduced number of parameters to estimate.

Three penalty functions for regularization were presented to extend the pre-existing *ℓ*_1_ penalty: The Adaptive Lasso, the Elastic Net and the non-convex *ℓ*_*q*_ penalty. All of them include one additional parameter which can be called *deformationd*. The limit case *d*=0 represents the *ℓ*_1_ method that has already been applied to the Systems Biology settings. When utilizing Adaptive Lasso or *ℓ*_*q*_, both, specificity and accuracy of the classification of cell-type specific parameters could be significantly increased for a realistic toy model involving Hill kinetics. The sensitivity remained unchanged in more than 50*%* of all runs. The Elastic Net did not show relevant improvements while being sensitive with respect to the deformation *d*. The difference between *ℓ*_*q*_ and Adaptive Lasso is significantly in favour of the former, whereas the total gain in accuracy is minor.

In theory, the dependence on *d* has to be assessed for each model individually. The results indicate that *ℓ*_*q*_ is the most robust method with respect to the predicted cell-type specific properties. Scanning over a range of admissible deformations allows to find alternative parsimonious models and to check the proposed models for multiple deformation types and strengths. Especially for *ℓ*_*q*_ it does however not seem necessary to scan over all deformations to simply regularize a model. A choice of, for instance, *q*=0.8 seemed viable for all models examined so far. The final parsimonious model is mostly non-unique among different deformations. Scanning all deformations can be regarded as an additional possibility to find hidden alternatives. Further statistical criteria such as the Bayesian Information Criterion might be employed to evaluate the most common final results.

The *ℓ*_*q*_ penalty function yielded the most reliable predictions for the simulated data. It has to be taken into account nevertheless that this method encounters issues with multiple minima whenever the penalty strength is sufficiently strong. Theoretically, the penalized objective function always has a (local) optimum if any fold-change is zero. Our method to circumvent this problem is twofold: First, the diverging derivatives are cut off at a small threshold. This implies that the penalty is made convex around zero, so the additional minimum at zero disappears for sufficiently strong objective function gradients. Parsimonious models can be found without this cut-off, but it renders the procedure more robust against log-fold-changes initialized as zero or set to zero erroneously while scanning the penalty strengths. Second, the range of penalty strengths *λ* that is scanned over to find the parsimonious model is started at small *λ*. Then, the optimizer can neglect the optimum in zero if it is not global. When increasing *λ*, the optimizer stays in the previously found optimum without being trapped in zero. It is hence necessary to initialize subsequent penalized optimizations at the values estimated before. The methods implemented in *Data2Dynamics* regularize a system accordingly. In further research the effects of concave penalty functions with a bounded gradient at zero could be of interest.

The increased specificity induced by deforming *ℓ*_1_ penalties can be explained by symmetry breaking effects. Especially in the case of linear correlations, which frequently occur in biological models, e.g. due to linked phosphorylation and dephosphorylation rates, the additional deformation degree of freedom allows to have penalty level lines that are not aligned along the correlation. Then, the penalization can act in a way that is less influenced by the objective function to shrink the model. If no log-transformation was applied to parameters, no linear correlation would arise and *ℓ*_1_ would not encounter problems. However, several benchmarking assessments have found log-transformations to be advantageous [31–33]. Thus, they should be kept and linear correlations should be handled by methods like those presented in our manuscript.

If the original objective function exhibits non-identifiabilities for fold-changes that are linearly correlated, the *ℓ*_1_ penalty terms are aligned to a subspace of constant objective-function, so the sum of objective function and penalty is constant on some interval, too. Then, any point inside this interval could be selected during the optimization. Symmetry breaking effects might then lead to a random selection of either end-point of the interval. This, however, is not a pitfall of the extended regularization, but of the model or the available data for structural or practical non-identifiablities, respectively. The extended methods can hence not cure non-identifiabilities. In this case, a model reformulation or additional, informative data are required to achieve sensitivity improvements.

None of the methods presented in this paper was able to increase the sensitivity of its predictions on cell-type specific properties. It could hence be doubted whether any new penalty approach that extends *ℓ*_1_ is able to predict more true positives than original *ℓ*_1_ and all methods described above.

Here, we focused on the comparison of two cell types, whereas the approach could easily be extended to incorporate any number of cell-types undergoing the same biochemical process. The implementation provided in *Data2Dynamics* can be used for this purpose. Therefore, one cell-type of reference has to be selected and fold-changes must be defined relating the reference-type to all remaining cell-types. Parameters can also be grouped as described in the *Group Lasso* technique [34]. The number of parameters then grows linearly with the number of cell-types. However, recent benchmarking results show that the performance depends polynomially on the number of parameters [33]. It should be examined in how far the choice of the reference cell-type affects the outcome since non-reference cells cannot be compared among each other, but only with the reference type. An examination of multiple cell-type models should be the subject of further studies.

The presented methods can also be generalized to any other field of Lasso usage which allows for a priori assumptions on some parameter values, such as zero log-fold-changes in our case. Yet, they are most valuable in parameter estimation problems of non-linear systems, and where collinearity may arise. This comprises descriptions of chemical reactions, e.g. for biochemical rate-equations, as well as deep-learning networks.

## Conclusion

In summary, we demonstrated that using extended *ℓ*_1_ methods can lead to a more specific and accurate classification of cell-type differences. The non-convex *ℓ*_*q*_ penalty, e.g. for *q*=0.8, provided best and fast predictions albeit it leads to non-convex objective functions. In particular, the application to the JAK2/STAT5 model showed that scanning the additional deformation parameter of the new methods facilitates the detection of differences in cell kinetics between a healthy and malignant cells, which go beyond what was published before based on *ℓ*_1_. Extended *ℓ*_1_ methods as described in this manuscript could play a role in unraveling fundamental features that characterize for instance cancer cells, possibly leading to new therapeutic entities and treatments.

## Data Availability

The regularization functions are available as MATLAB routines within the open-source modelling framework *Data2Dynamics*. The DREAM6 network with the parameters used for the presented simulations and all biological data used from the JAK2/STAT5 signalling pathway are also included in this software package.

## Abbreviations

AIC: Akaike information criterion
BIC: Bayesian information criterion
CFU-E: Colony forming unit-erythroid
CISH: cytokine-inducible SH2-containing protein
CISHRNA: Ribonucleic acid of CISH
DREAM6: 6th Dialogue for reverse engineering assessment and methods challenge
EPO: Erythropoietin
JAK2: Janus kinase 2
Lasso: Least absolute shrinkage and selection operator
LRT: Likelihood ratio test
ML: Maximum likelihood
mRNA: Messenger ribonucleic acid
ODE: Ordinary differential equation
ROC: Receiver operating characteristics
SOCS3: Suppressor of cytokine signalling 3
STAT5: Signal transducer and activator of transcription 5

## References

[1] Kitano H (2002). Systems Biology: A Brief Overview. Science.

[2] Thompson ML (1978). Selection of Variables in Multiple Regression: Part I. A Review and Evaluation. Int Stat Rev.

[3] Hocking RR, Leslie RN (1967). Selection of the Best Subset in Regression Analysis. Technometrics.

[4] Tikhonov AN (1963). On the Solution if Ill-Posed Problems and the Method of Regularization. Doklady Akademii Nauk SSSR.

[5] Phillips DL (1962). A Technique for the Numerical Solution of Certain Integral Equations of the First Kind. J ACM.

[6] Franklin JN (1974). On Tikhonov’s Method for Ill-Posed Problems. Math Comput.

[7] Hoerl AE, Kennard RW (2000). Ridge Regression: Biased Estimation for Nonorthogonal Problems. Technometrics.

[8] Tibshirani R (1996). Regression Shrinkage and Selection via the Lasso. J R Stat Soc Ser B.

[9] Vidaurre D, Bielza C, Larrañaga P (2013). A Survey of L1 Regression. Int Stat Rev.

[10] Zou H (2006). The Adaptive Lasso and Its Oracle Properties. J Am Stat Assoc.

[11] Zou H, Hastie T (2005). Regularization and Variable Selection via the Elastic Net. J R Stat Soc Ser B.

[12] Tuia D, Flamary R, Barlaud M (2016). Nonconvex Regularization in Remote Sensing. IEEE Trans Geosci Remote Sens.

[13] Merkle R, Steiert B, Salopiata F, Depner S, Raue A, Iwamoto N (2016). Identification of Cell Type-Specific Differences in Erythropoietin Receptor Signaling in Primary Erythroid and Lung Cancer Cells. PLoS Comput Biol.

[14] Steiert B, Timmer J, Kreutz C (2016). L1 Regularization Facilitates Detection of Cell Type-Specific Parameters in Dynamical Systems. Bioinformatics.

[15] Bachmann J, Raue A, Schilling M, Böhm ME, Kreutz C, Kaschek D (2011). Division of Labor by Dual Feedback Regulators Controls JAK2/STAT5 Signaling Over Broad Ligand Range. Mol Syst Biol.

[16] Raue A, Kreutz C, Maiwald T, Bachmann J, Schilling M, Klingmüller U (2009). Structural and Practical Identifiability Analysis of Partially Observed Dynamical Models by Exploiting the Profile Likelihood. Bioinformatics.

[17] Raue A, Schilling M, Bachmann J, Matteson A, Schelker M, Kaschek D (2013). Lessons Learned from Quantitative Dynamical Modeling in Systems Biology. PloS ONE.

[18] Raue A, Steiert B, Schelker M, Kreutz C, Maiwald T, Hass H (2015). Data2Dynamics: A Modeling Environment Tailored to Parameter Estimation in Dynamical Systems. Bioinformatics.

[19] Kreutz C, Bartolome Rodriguez MM, Maiwald T, Seidl M, Blum HE, Mohr L (2007). An Error Model for Protein Quantification. Bioinformatics.

[20] Magnus JR (1978). Maximum Likelihood Estimation of the GLS Model with Unknown Parameters in the Disturbance Covariance Matrix. J Econ.

[21] Murphy SA, van der Vaart AW (2000). On Profile Likelihood. J Am Stat Assoc.

[22] Venzon DJ, Moolgavkar SH (1988). A Method for Computing Profile-Likelihood-Based Confidence Intervals. J R Stat Soc: Ser C.

[23] Wilks SS (1938). The Large-Sample Distribution of the Likelihood Ratio for Testing Composite Hypotheses. Ann Math Stat.

[24] Akaike H (1974). A new look at the statistical model identification. IEEE Trans Autom Control.

[25] Schwarz G (1978). Estimating the Dimension of a Model. Ann Stat.

[26] Rockafellar RT (1979). Directionally Lipschitzian Functions and Subdifferential Calculus. Proc Lond Math Soc.

[27] Becker V, Schilling M, Bachmann J, Baumann U, Raue A, Maiwald T (2010). Covering a broad dynamic range: information processing at the erythropoietin receptor. Science.

[28] Hass H, Masson K, Wohlgemuth S, Paragas V, Allen JE, Sevecka M (2017). Predicting ligand-dependent tumors from multi-dimensional signaling features. npj Syst Biol Appl.

[29] Maiwald T, Hass H, Steiert B, Vanlier J, Engesser R, Raue A (2016). Driving the Model to Its Limit: Profile Likelihood Based Model Reduction. PLoS ONE.

[30] Steiert B, Raue A, Timmer J, Kreutz C (2012). Experimental Design for Parameter Estimation of Gene Regulatory Networks. PloS ONE.

[31] Kreutz C (2016). New Concepts for Evaluating the Performance of Computational Methods. IFAC-PapersOnLine.

[32] Villaverde AF, Fröhlich F, Weindl D, Hasenauer J, Banga JR (2018). Benchmarking optimization methods for parameter estimation in large kinetic models. Bioinformatics.

[33] Hass H, Loos C, Alvarez ER, Timmer J, Hasenauer J, Kreutz C. Benchmark Problems for Dynamic Modeling of Intracellular Processes. Bioinformatics. 2019;btz020. 10.1093/bioinformatics/btz020 PMC6735869 30624608

[34] Yuan M, Lin Y (2006). Model Selction and Estimation in Regression with Grouped Variables. J R Stat Soc Ser B.

